# Iron Overload Protects from Obesity by Ferroptosis

**DOI:** 10.3390/foods10081787

**Published:** 2021-08-01

**Authors:** Wan Ma, Li Jia, Qingqing Xiong, Huahua Du

**Affiliations:** Key Laboratory of Molecular Animal Nutrition, Ministry of Education, College of Animal Sciences, Zhejiang University, Hangzhou 310058, China; 11917002@zju.edu.cn (W.M.); 3150100159@zju.edu.cn (L.J.); 21917019@zju.edu.cn (Q.X.)

**Keywords:** iron overload, ferroptosis, glucolipid metabolism, obesity

## Abstract

Dysregulation in iron metabolism is associated with obesity, type 2 diabetes, and other metabolic diseases, whereas the underlying mechanisms of imbalanced glycolipid metabolism are still obscure. Here, we demonstrated that iron overload protected mice from obesity both with normal diets (ND) or high-fat diets (HFD). In iron-overload mice, the body fat was significantly decreased, especially when fed with HFD, excessive iron mice gained 15% less weight than those without iron supplements. Moreover, glucose tolerance and insulin sensitivity were all significantly reduced, and hepatic steatosis was prevented. Furthermore, these mice show a considerable decrease in lipogenesis and lipidoses of the liver. Compared with control groups, iron treated groups showed a 79% decrease in the protein level of Perilipin-2 (PLIN2), a protein marker for lipid droplets. These results were consistent with their substantial decrease in adiposity. RNA-seq and signaling pathway analyses showed that iron overload caused ferroptosis in the liver of mice with a decrease in GPX4 expression and an increase in Ptgs2 expression, resulting in a high level of lipid peroxidation. Overall, this study reveals the protective function of iron overload in obesity by triggering the imbalance of glucolipid metabolism in the liver and highlights the crucial role of ferroptosis in regulating lipid accumulation.

## 1. Introduction

Iron is required for life to survive and must be orchestrated in the body. An imbalance of iron homeostasis can result in many undesirable consequences. In several chronic metabolic disorders, including nonalcoholic fatty liver disease (NAFLD) [1], type 2 diabetes mellitus (T2DM) [2], and cardiovascular diseases [3], iron overload affects their clinical processes. Iron overload is a state in which the body has too much iron, causing iron to accumulate in organs such as the liver, pancreas, and heart, leading to these crucial organs’ malfunction [4]. The liver is in charge of iron absorption, utilization, storage, and recycling. Excessive hepatic iron can cause liver injury via the formation of reactive oxygen species (ROS), which increases the risk for cirrhosis and liver cancer [5].

Systemic iron insufficiency is one of the pathologic statuses seen in obesity, which is accompanied by hypoferremia. Obesity decreases the amount of accessible intracellular iron and induces inflammatory levels, which enhances the expression of hepcidin [6]. Hepcidin inhibits iron efflux by internalizing and then degrading the iron exporter called ferroportin 1, which is located on the exterior of duodenal enterocytes and hepatocytes. Iron has recently been found as a critical regulator of energy metabolism, particularly in adipose tissue [7]. Adipose tissue helps to dynamically maintain the balance of glucose and lipid metabolism in the body. In adipose tissue, iron deficiency has both been related to insulin resistance and adipokine secretion [8]. Excessive iron is also considered to have a role in a variety of obesity-related disorders, including hepatic steatosis and T2DM [5,9].

Previously, we demonstrated that high-iron diets would decrease the body weight gain in a mouse model of T2DM built by C57BLKSJ-lepr (db/db) mice with an increasing iron concentration in the serum, indicating that excess iron plays a diverse role in the process of diabetes and obesity [10]. As a regulatory center of glucose and lipid metabolism, hepatocytes are responsible for maintaining steady glucose and lipoprotein concentrations in the blood. The mechanism by which iron excess in the liver affects glucolipid metabolism, however, is yet unknown. This study set out to see if iron overload might impact the development of obesity and the chronic conditions that come with it. 

## 2. Materials and Methods

### 2.1. Animal Experiments

The Zhejiang University Committee on Experimental Animal Care authorized the mouse studies (ZJU2015-447-09). Three-week-old male C57BL/6 mice (Nanjing Biomedical Research Institute, Nanjing, China) were randomly divided into ND (based on the research diets D12450J formulation [11] with its iron content is about 9 mg/kg), iron treated (ND + Fe), HFD (based on the research diets D12492 60 kcal% fat formulation [11] with its iron content is about 9 mg/kg), and iron treated HFD (HFD + Fe) groups (8 mice per group). Mice were fed with ND or HFD for 12 weeks. Iron-treated mice were given 120 μg/g body weight of iron dextran (Pharmacosmos, Denmark) intraperitoneally every other week for 12 weeks, starting at 4 weeks old. In contrast, iron-untreated mice were injected intraperitoneally with saline. Mice were housed under standardized temperature conditions (22 ± 1 °C) with a 12 h light-dark cycle and kept freely with access to feed and water. Every week, the body weights and average feed consumption were recorded. Fasting was performed overnight before each sampling. Mice were sacrificed by cervical dislocation at the end of the experiment. Livers and adipose tissue were rapidly dissected, fixed in 4% paraformaldehyde solution for histological analysis, or frozen quickly in liquid nitrogen and kept at −80 °C until analysis.

### 2.2. Serum and Hepatic Biochemical Assays

For blood glucose determination, blood samples were harvested from the tail vein and measured with a Contour TS Meter Glucometer (Bayer Diagnostics, Leverkusen, Germany). For other measurements, blood was taken from their hearts after the mice were anesthetized. Blood was then centrifuged at 1000× *g* at 4 °C to get the supernatant, which was kept at −80 °C until further detection. Serum iron was measured using a serum iron assay kit, and hepatic iron content was detected by a tissue iron assay kit (Jiancheng Biology, Nanjing, China). Hepatic malondialdehyde (MDA) and glutathione (GSH) content were measured using kits (Beyotime, Haimen, China). Serum adiponectin, triglycerides, cholesterol, and insulin levels were quantified by ELISA kits (MBbiology, Yancheng, China) on the basis of the manufacturer’s instructions.

### 2.3. Glucose and Insulin Tolerance Tests

Mice were fasted overnight before the studies. Mice were given a 1 g/kg body weight intraperitoneal injection of glucose for the glucose tolerance test, and were given 0.75 IU insulin/kg body weight intraperitoneally for the insulin tolerance test [12]. The glucometer was used to measure blood glucose levels, and areas under the curve were calculated using GraphPad Prism 8.0 (Graphpad Software, San Diego, CA, USA).

### 2.4. Histological Analysis of the Liver

For iron staining, samples were embedded by paraffin and cut into slices (2 μm) before being stained with Prussian blue. Frozen slices (10 μm) of the liver were fixed with 10% formalin for 10 min and then stained with Oil Red O (Sigma-Aldrich, St. Louis, MO, USA) at 60 °C for 10 min to identify lipid accumulation. Three images were chosen at random fields from a portion of each mice, and then captured by a light microscope equipped with a digital camera (Nikon, Eclipse Ci, Tokyo, Japan).

### 2.5. Real-Time Polymerase Chain Reaction (PCR) Analysis

The PrimeScript RT kit (Takara, Shiga, Japan) was applied to reverse transcribe total RNA extracted from liver tissues into cDNA. The ABI 7500 Real-time PCR System and FastStart Universal SYBR Green Master (ROX; Roche, Basel, Switzerland, USA) were used to perform real-time PCR (Applied Biosystems, Waltham, MA, USA). The fold difference in gene expression was calculated using the 2^−ΔΔCt^ method, using β-actin mRNA as control. All reactions were carried out at least three times, and specificity was tracked using a melting curve analysis. The sequences of the primers are listed in Table 1.

### 2.6. Western Blot Analysis

Protein samples (20 µg) of mouse liver were applied to 8% to 12% SDS-PAGE for electrophoretic separation and immunoblotting. The following were the primary antibodies used in the Western blot assay: anti-p-HSL (#4126; Cell Signaling Technology, Danvers, MA, USA), anti-HSL (#4107, Cell Signaling Technology), anti-PGC1α (#54481, Abcam, Cambridge, UK), anti-PPARγ (#7273, Santa Cruz Biotechnology, Santa Cruz, CA, USA), anti-C/EBPα (#8178, Cell Signaling Technology), anti- PLIN2 (#108323, Abcam), anti-PCK1 (#70358, Abcam), anti-G6Pc (#83690, Abcam), anti-IRβ (#3025, Cell Signaling Technology), anti-IRS-1 (#3407, Cell Signaling Technology), anti-PI3K (#4257, Cell Signaling Technology), anti-p-AKT (#9271, Cell Signaling Technology), anti-AKT (#9272, Cell Signaling Technology), anti-CAT (#ER40125, HuaBio, Hangzhou, China), anti-SOD2 (#ET1701-54, HuaBio), anti-COX2 (#12282, Cell Signaling Technology), anti-GPX4 (#125066, Abcam), anti-FtH (#81444, Abcam), anti-xCT (#37185, Abcam), and anti-β-actin (#8457, Cell Signaling Technology). Peroxidase-conjugated goat anti-rabbit IgG (BL003A, Biosharp, Hefei, China) and goat anti-mouse IgG (BL001A, Biosharp) were applied as secondary antibodies.

### 2.7. RNA-Seq Analysis

Novogene Bioinformatics Technology Co., Ltd performed the RNA-seq analysis (Beijing, China). Following the manufacturer’s instructions, sequencing libraries were generated from 1 µg total RNA using the NEBNext^®^ UltraTM RNA Library Prep Kit for Illumina® (NEB, San Diego, CA, USA). Then, the libraries were quantified and pooled. The Illumina NovaSeq 6000 platform was used to sequence a paired-end cDNA library, consisting of paired-end 150 base pair reads. For assessing the quality of sequencing data, FastQC (version 0.11.2) was utilized. StringTie was used to calculate the transcript gene expression values (version 1.3.3b, The Center for Computational Biology at Johns Hopkins University, Baltimore, MD, USA). Gene lengths and read counts assigned to this gene were used to determine the FPKM. The DESeq2 R package (version 1.16.1, Bioconductormanufacture) was used to perform differential expression analysis of two groups. If *p* < 0.05, |log2FC| > 0, genes were deemed significantly differentially expressed. The cluster Profiler R package was applied to perform gene ontology (GO) and Kyoto Encyclopedia of Genes and Genomes (KEGG) enrichment. GO functional enrichment analysis and KEGG pathway enrichment were shown in bubble diagrams. The calibration value of P value is Q value. The color indicates enrichment Q value. Simply put, darker color represents a smaller Q value. The *X*-axis shows enrichment ratio, and the Y-axis represents GO Term or KEGG Pathway. The size of bubbles represents the number of genes annotated to a GO Term or KEGG Pathway.

### 2.8. Statistical Analyses

All assays were performed at least three times, and the results are shown as the means ± SEMs. SPSS 20 (IBM, Armonk, NY) and GraphPad Prism 8.0 (GraphPad, San Diego, CA, USA) were used to conduct the statistical analysis. The unpaired two-tailed Student’s t test was applied to compare the differences between the two groups. The significance level was set at *p* < 0.05. Statistically significant differences are displayed as * *p* < 0.05, ** *p* < 0.01, and *** *p* < 0.001.

## 3. Results

### 3.1. Iron Overload Protects against HFD-Induced Obesity

**Figure 1:**
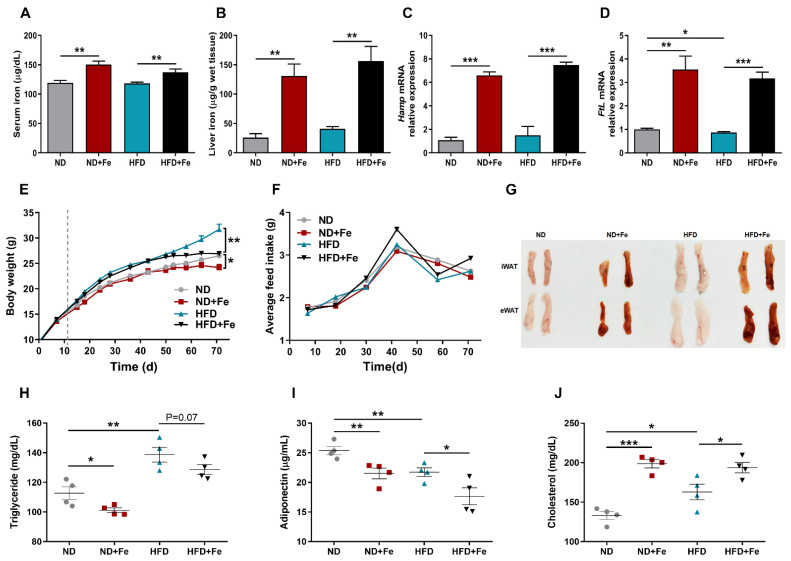
Iron overload protects against HFD-induced obesity. (**A**) Fasting iron content in the serum; (**B**) Hepatic iron content; (**C**,**D**) Relative gene expression of hepcidin (Hamp) and ferritin L (FtL) in liver samples; (**E**) Body weight; (**F**) Average feed intake; (**G**) Representative images of iWAT and eWAT in mice; (**H**) Fasting serum levels of triglycerides; (**I**) Fasting serum levels of adiponectin; (**J**) Fasting serum levels of cholesterol. ND (*n* = 6), ND + Fe (*n* = 7), HFD (*n* = 7), and HFD + Fe (*n* = 8). The values are the means ± SEMs. * *p* < 0.05, ** *p* < 0.01, *** *p* < 0.001. Significant differences between two groups were analyzed with the two-tailed Student’s *t* test.

To determine whether iron overload has a major impact on the development of obesity, we fed mice with a HFD for 12 weeks. Mice were given iron dextran intraperitoneally every other week for 12 weeks to establish the iron-overloaded model. Iron-treated mice had higher iron levels in the serum and tissue than iron-untreated mice (Figure 1A,B). Compared with iron-untreated mice, the iron-injected mice with the higher systemic iron levels increased the mRNA expression of hepcidin and ferritin, which is consistent in line with previous studies (Figure 1C,D). Interestingly, the body weight of iron-treated mice was significantly lower than that of controls after 12 weeks both fed with ND and HFD (Figure 1E). Mice in HFD + Fe group gained 15% less weight than those in the HFD group with no significant difference in their average feed intake (Figure 1F). It showed that the lowered weight gain observed was not related to a lower appetite. Moreover, the appearance of inguinal white adipose tissue (WAT) and epididymal WAT was much smaller and darker following iron administration (Figure 1G). Further, lipid levels in the serum are commonly changed in obesity. The analysis of lipid levels revealed a significant reduction in triglyceride (Figure 1H) and adiponectin content (Figure 1I) and increased cholesterol content (Figure 1J) in the serum.

### 3.2. Reduced Adipocyte Hypertrophy Due to Iron Overload

**Figure 2:**
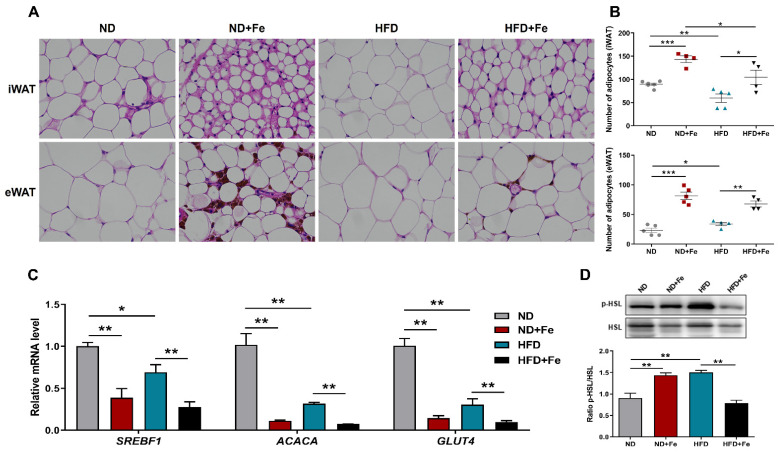
Reduced adipocyte hypertrophy due to iron overload. (**A**) Representative images of iWAT and eWAT fat pad sections stained with H&E; (**B**) Number of adipocytes of iWAT and eWAT; (**C**) Relative gene expression of SREBF1, ACACA and GLUT4 in eWAT samples; (**D**) Western blot analysis of phosphor-HSL and total HSL protein expression and their quantification in liver samples. ND (*n* = 6), ND + Fe (*n* = 7), HFD (*n* = 7), and HFD + Fe (*n* = 8). The values are the means ± SEMs. * *p* < 0.05, ** *p* < 0.01, *** *p* < 0.001. Significant differences between two groups were analyzed with the two-tailed Student’s *t* test.

We undertook histological analyses of WAT to better explain the observed decrease in fat accumulation in iron-overload mice after HFD. Both inguinal WAT and epididymal WAT analysis revealed that the size of adipocytes was significantly reduced in iron injected mice compared with controls (Figure 2A). Accordingly, the decrease in the volume of adipocyte was accompanied by an increasing number of adipocytes per unit area (Figure 2B). Furthermore, the expression levels of genes implicated in adipocyte differentiation were examined, such as sterol regulatory element binding-protein 1 (*SREBF1*), acetyl-CoA carboxylase alpha (*ACACA*) and glucose transport 4 (*GLUT4)*, were down-regulated in WAT of iron-treated mice (Figure 2C). We investigated the phosphorylation state of hormone-sensitive lipase (HSL) further since it is largely controlled by post-translational processes rather than at the mRNA level. In mice fed with HFD, Western blot analysis indicated a substantial rise in the ratio of phospho-HSL and total HSL levels, which was significantly reduced after iron treatment (Figure 2D). Interestingly, HSL phosphorylation was similarly increased in iron-treated mice without HFD feeding.

### 3.3. Decreased Lipidoses in the Liver of Iron-Overloaded Mice

**Figure 3:**
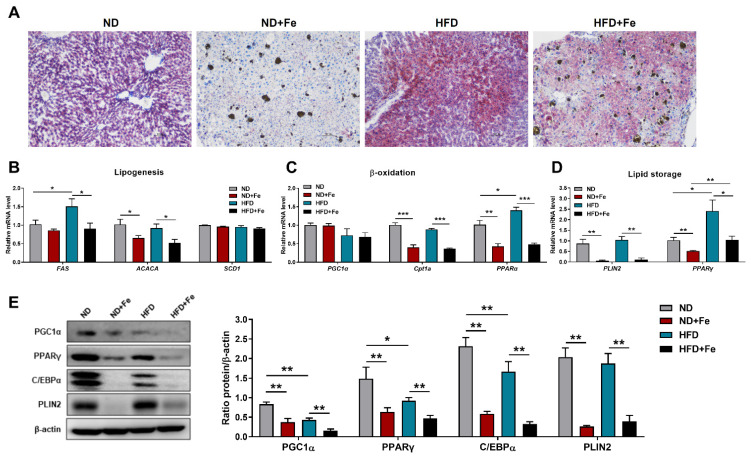
Decreased lipidoses in the liver of iron-overloaded mice. (**A**) Representative Oil Red O staining of liver slices; (**B**) Relative expression levels of genes related to lipogenesis (FAS, ACACA, SCD1) in liver samples; (**C**) Relative expression levels of genes related to Cpt1a, PPARα) in liver samples; (**D**) Relative expression levels of genes related to lipid storage (PLIN2, PPARγ) in liver samples; (**E**) Western blot analysis of PGC1α, PPARγ, CEBP/α, PLIN2 protein expression and their quantification in liver samples. ND (*n* = 6), ND + Fe (*n* = 7), HFD (*n* = 7), and HFD + Fe (*n* = 8). The values are the means ± SEMs. * *p* < 0.05, ** *p* < 0.01, *** *p* < 0.001. Significant differences between two groups were analyzed with the two-tailed Student’s *t* test.

We further analyzed whether iron overload alleviates hepatic steatosis, a prevalent disease commonly linked to obesity. Oil Red O staining of mouse liver slices showed a marked reduction in lipid accumulation in the iron-treated groups compared to their control counterparts (Figure 3A). We assessed the level of lipid synthesis, fatty acid oxidation, and lipid storage by the mRNA expression of related genes to see if the observed decrease in lipid content was related to changes in hepatic lipid metabolism. After exposing mice to HFD, the expression of fatty acid synthase (*FAS*) was significantly increased. However, there was no effect on *ACACA* or stearoyl-CoA desaturase 1 (*Scd1*), which are both related to lipogenesis (Figure 3B). Notably, iron treatment suppressed the mRNA abundance of *FAS* and *ACACA*. Additionally, the mRNA levels of peroxisome proliferator-activated receptor γ coactivator-1 α (*PGC1α*) and carnitine palmitoyl transferase-1a (*Cpt1a*), which are fatty acid β-oxidation related genes, showed no significant difference in HFD mice when compared to the control group, excluding peroxisome proliferator-activated receptor α (*PPARα*). In comparison, the transcript levels of *Cpt1a* and *PPARα* were both repressed by iron treatment (Figure 3C). Furthermore, the mRNA levels of *PLIN2*, a vital regulator of lipid droplet growth [13], and peroxisome proliferated activated receptor γ (*PPARγ)*, a key driver of hepatic triglyceride storage [14], were both significantly (*p* < 0.01) decreased upon iron administration (Figure 3D). Western blot analysis showed that iron injection dramatically reduced the expression level of three main lipid metabolism regulators, including PGC1α, PLIN2, and PPARγ (Figure 3E). Compared with the ND group, the expression level of PLIN2 in the ND + Fe group decreased 87% and compared with the HFD group, the expression level of PLIN2 in the HFD + Fe group dropped 79%. This reduction was consistent with a dramatic decrease in the expression level of CCAAT/enhancer binding protein α (C/EBPα), a protein necessary for adipogenesis [15] and tightly associated with general liver lipid metabolism (Figure 3E). Taken together, these findings suggested a protective role of excessive iron supplementation in the process of lipid dysregulation in the liver of mice, especially with diet-induced obesity.

### 3.4. Decreased Insulin Sensitivity in Iron-Overloaded Mice

**Figure 4:**
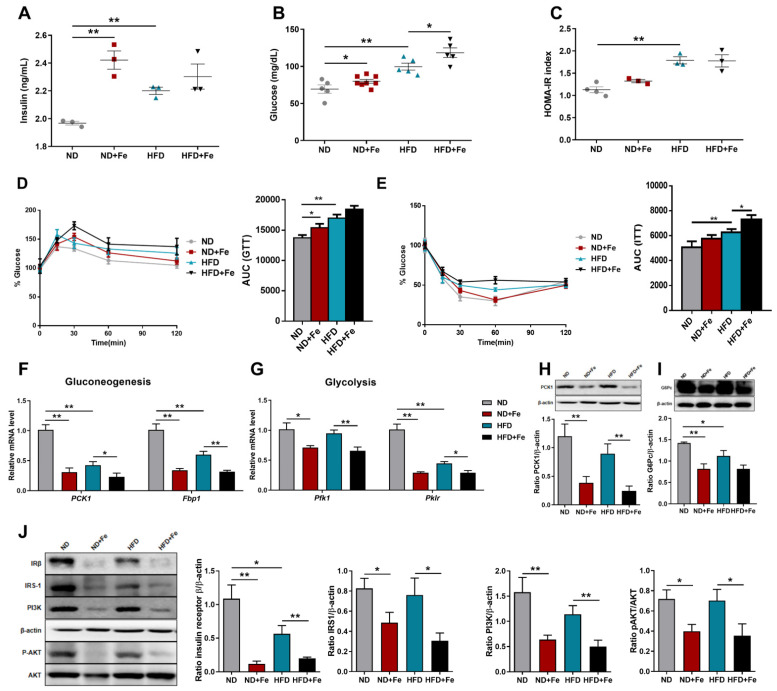
Deleterious insulin sensitivity in iron-overloaded mice. (**A**) Fasting insulin concentration in the serum; (**B**) Fasting blood glucose concentration; (**C**) HOMA-IR index; (**D**) Glucose tolerance test after fasting overnight; (**E**) Insulin tolerance test after fasting overnight. AUC, area under curve; (**F**) Relative expression levels of genes related to gluconeogenesis (PCK1, Fbp1) in the liver; (**G**) Relative expression levels of genes involved in glycolysis (Pfk1, Pklr) in the liver; (**H**,**I**) Western blot analysis of PCK1 and G6Pc protein expression and its quantification in liver samples; (**J**) Western blot analysis of IRβ, IRS-1, PI3K, p-AKT and total AKT protein expression and their quantification in liver samples. ND (*n* = 6), ND + Fe (*n* = 7), HFD (*n* = 7), and HFD + Fe (*n* = 8). The values are the means ± SEMs. * *p* < 0.05, ** *p* < 0.01. Significant differences between two groups were analyzed with the two-tailed Student’s *t* test.

The dramatic resistance to the development of the obese phenotype exhibited by iron-overload mice triggered us to assess whether iron overload affected glucose homeostasis. Surprisingly, higher plasma insulin (Figure 4A) and fasting blood glucose (Figure 4B) levels were both found in iron-overloaded mice compared to control groups. HOMA-IR index, widely used as an insulin resistance index, was increased significantly in HFD-fed mice, and iron administration could not reverse the trend (Figure 4C), suggesting the possibility of decreased insulin sensitivity after iron supplementation. To further explore glucose homeostasis under iron overload conditions, the mice were given glucose intraperitoneally to perform glucose tolerance tests. These experiments revealed a significantly worse glucose clearance in iron-injected mice compared with control mice (Figure 4D). Similarly, blood glucose levels increased in iron-treated mice that performed insulin injections (Figure 4E), suggesting that these mice had a lower insulin sensitivity, which was consistent with their higher insulin level. These findings support the idea that iron overload causes insulin resistance in the body [16], which resulted in dysregulation of glucose response. We then determined to see if the down-regulation in the expression levels of genes involved in gluconeogenic and glycolytic enzymes could contribute to the impaired glucose response caused by iron overload. Analysis of the mRNA expression levels of key genes, such as *PCK1*, *Fbp1*, *Pfk1*, and *Pklr*, were significantly down-regulated in iron-treated mice compared to controls (Figure 4F,G). Both phosphoenolpyruvate carboxykinase 1 (PCK1) and glucose-6-phsophatase catalytic (G6Pc) proteins exhibited a significant decrease with iron administration (Figure 4H,I) consistent with the observed decreases in gluconeogenesis. Moreover, the insulin receptor is a critical component of insulin signal transduction, and its malfunction is thought to be the fundamental cause of T2DM [17]. The protein levels of insulin receptor β (IRβ) and IR substrate 1 (IRS-1) were both decreased in the liver of iron-treated groups compared to control groups (Figure 4J). The phosphatidylinositol 3-kinase (PI3K)/ protein kinase B (AKT) pathway is regarded as regulating glucose metabolism. Both PI3K protein and the ratio of p-AKT levels to total AKT protein were significantly decreased in iron-treated mice compared to controls (Figure 4J). These results suggested that impaired insulin sensitivity observed in iron-treated mice may be due to the reduced insulin signaling level. Altogether, in iron-overloaded mice, resistance to fat accumulation not only led to insulin resistance but also to dysregulation of glucose and lipid metabolism.

### 3.5. Enriched Ferroptosis and Fatty Acid Metabolism Pathways by Excess Iron

**Figure 5:**
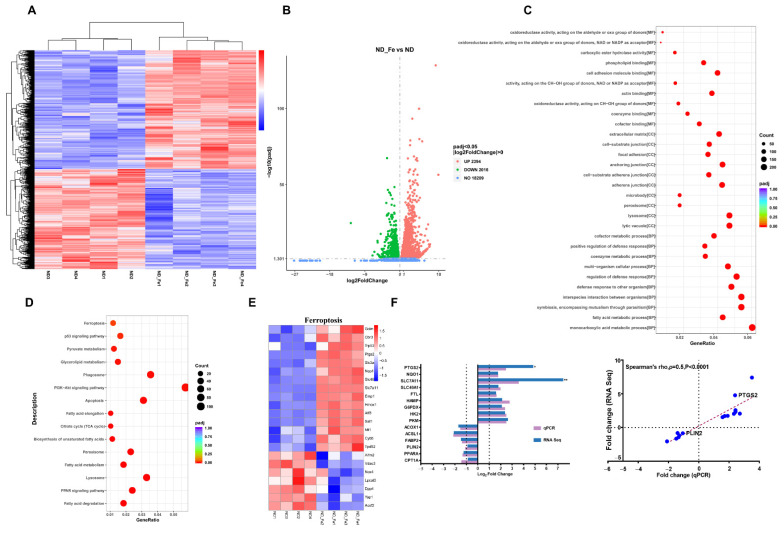
Enriched ferroptosis and fatty acid metabolism pathways by excess iron. (**A**) Cluster analysis of differentially expressed genes; (**B**) DEGs among ND and ND + Fe groups were showed by volcanic maps. After log2 conversion, the X-axis represents the difference multiples, and after log10 conversion, the Y-axis displays the significant value. (P_adj_ < 0.05 & Abs (Log2 fold change) > 0); (**C**,**D**) GO functional enrichment analysis and KEGG pathway enrichment are shown in a bubble diagram; (**E**) Heatmap showing relative expression of chosen ferroptosis regulated genes from DEGs. Only genes with P < 0.05 are shown; (**F**) qPCR validation of RNA-seq findings and correlation analysis of RNA-seq and qPCR. Expression profiles of RNA-seq data and qPCR data of chosen genes were compared within the same samples. The results are provided as Log2 values of the fold change and represent mean values. ND (*n* = 4), ND + Fe (*n* = 4).

To identify the underlying mechanism of alterations of glucolipid metabolism under iron overload conditions in the liver, an RNA sequence analysis of liver tissue from ND and ND + Fe mice was performed (Figure 5A). To characterize the functional consequences of gene expression changes caused by iron supplementation, different expression gene sets (DEGs) were identified using the following criteria: |log2FoldChange| > 0 and P_adj_ < 0.05. As a result, 4410 DEGs were identified, of which 2394 DEGs were upregulated and 2016 DEGs were downregulated (Figure 5B). GO enrichment analysis of the genes induced by iron overload revealed pronounced alterations in the oxidoreductase activity and lysosome (Figure 5C), suggesting that redox reactions and programmed cell death may occur in the progression of iron-overloaded mice. It also showed that fatty acid metabolic and monocarboxylic acid metabolic-related genes were enriched in the regulation of fatty acid oxidation and metabolic intermediates (Figure 5C). Functional enrichment analysis using KEGG pathways showed several significantly changed DEGs, including ferroptosis and fatty acid metabolism-related pathways (Figure 5D). Ferroptosis-related genes among DEGs were listed, among which *Slc7a11* and *Hmox1* were more than 10-fold more upregulated in the liver after iron treatment (Figure 5E). Finally, DEG mRNA expression level was validated by qRT-PCR and the results were consistent with those from RNA-seq (Figure 5F).

### 3.6. Induced Ferroptosis in the Liver of Iron Overload Mice

**Figure 6:**
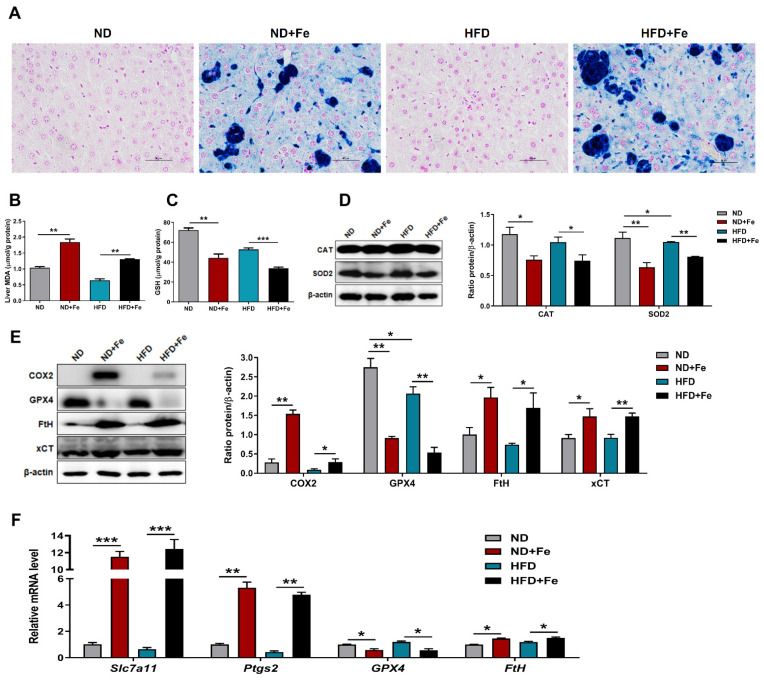
Induced ferroptosis in the liver of iron overload mice. (**A**) Representative images of liver sections stained with Perl’s blue; (**B**) Hepatic MDA content; (**C**) Hepatic GSH content; (**D**) Western blot analysis of CAT, SOD2 protein expression and their quantification in liver samples; (**E**) Western blot analysis of ferroptosis related protein (COX2, GPX4, FtH, xCT) expression and their quantification in liver samples; (**F**) Relative expression levels of genes related to ferroptosis (Slc7a11, Ptgs2, GPX4, FtH) in liver. ND (*n* = 6), ND + Fe (*n* = 7), HFD (*n* = 7), and HFD + Fe (*n* = 8). The values are the means ± SEMs. * *p* < 0.05, ** *p* < 0.01, *** *p* < 0.001. Significant differences between two group were analyzed with the two-tailed Student’s *t* test.

Ferroptosis is a newly found method of cell death caused by iron-dependent lipid peroxidation [18]. Ferroptosis is characterized by increased intracellular iron content, increased lipid peroxidation at the cell membrane, as well as depletion of reduced nicotinamide adenine dinucleotide phosphate. Currently, three biomarkers have been used for identifying the occurrence of ferroptosis: boosted lipid peroxidation levels, increased gene expression level of Ptgs2, and decreased glutathione peroxidase 4 (GPX4) expression [19,20]. In this study, Perl’s staining of liver sections revealed obviously increased iron deposition in hepatocytes after iron injection (Figure 6A). MDA, as a measurement of lipid peroxidation in tissues, were raised in the liver of iron-overloaded mice (Figure 6B). Consistent with this, the content of GSH, one of the main antioxidants, was also significantly reduced, suggesting that extra protein may help maintain redox balance during iron overload (Figure 6C). The Fenton reaction transits from ferric to ferrous iron and produces ROS, harming cellular membranes and nuclei. Western blot analysis showed that the expression of antioxidant enzymes, such as catalase (CAT) and superoxide dismutase 2 (SOD2), were both reduced after iron treatment, which might be associated with an increase in ROS (Figure 6D). Furthermore, the expression of xCT (*Slc7a11*), which is the main part of the glutamate/cystine antiporter system X_C_^-^ and prevents lipid peroxidation-induced cell death [18], was significantly upregulated in iron-treated mice at the mRNA and protein levels, as well as those of cyclooxygenase 2 (COX2, Ptgs2) and ferritin heavy chain (FtH) (Figure 5E,F). Besides, iron treatment significantly decreased hepatic GPX4 protein and mRNA levels (Figure 5F), which encode lipid peroxidation scavenging proteins that are GSH-dependent or independent. Overall, these findings suggested that iron overload causes ferroptosis in both ND and HFD mice.

## 4. Discussion

Our results demonstrated that iron overload was associated with decreased weight in both mice fed ND and HFD diets. Iron-overloaded mice showed a protection against the formation of body fat with reduced concentration of triglyceride in serum and adipocyte hypertrophy of WAT. Associations between hepatic iron and the development of steatosis have been discovered in rodent models of fatty liver disease. Evidence showed that steatosis that developed in rats fed a HFD diet resulted from activation of SREBF-1 and oxidative stress [21]. In contrast, mice with hereditary iron deficit were resistant to obesity when fed a HFD diet [22]. In this study, compared to those of iron-untreated mice, key genes associated with adipocyte differentiation, like *SREBF-1*, *ACACA*, and *GLUT4*, were all significantly down-regulated in the WAT of iron-treated mice. 

Dietary iron is absorbed by intestinal epithelial cells, whereas splenic iron is produced by macrophages and then flows straight to the liver via portal circulation. The liver is the key organ that regulates iron homeostasis in the body via the iron-regulatory hormone hepcidin. There is high metabolic demand for iron to carry out energy generation, biosynthesis, and detoxification in the liver [23]. In this study, iron overload prevented the development of hepatic steatosis by suppressing the expression of genes involved in hepatic lipogenesis and lipidoses. Increased tissue iron levels are strongly linked to NAFLD in human hereditary hemochromatosis (HH), an autosomal recessive disease in which the body stores too much iron that usually presents with mutations in the HFE gene [24]. Hepcidin knock-out mice with a phenotype of excessive hepatic iron could reverse the hepatic steatosis caused by HFD in HH mice [22]. However, iron overload also resulted in decreased glucose tolerance and increased insulin secretory capacity, processes associated with obesity and diabetes. Given the contradictory impacts of iron overload on diabetes risk, such as obesity protection yet impaired insulin sensitivity, further human mechanistic investigations are needed. Because of the lower insulin secretory capacity caused by hyperinsulinemia, it is less possible for obese people with HH to compensate for insulin resistance [25]. These findings further highlight the debate over the relative roles of insulin resistance and insulin secretion in the development of diabetes in HH [26].

To explore the underlying regulation mechanism, RNA-seq was performed in the mouse liver. RNA-seq analysis showed that fatty acid metabolism and the ferroptosis pathway were enriched. The first and most fully researched mechanism hypothesized to explain the hepatotoxicity of excessive iron was free radical-mediated hepatocellular damage [27], so the enrichment of the ferroptosis signaling pathway attracted our attention. Ferroptosis is a novel sort of cell death that occurs when GPX4 is inhibited by a reduction in GSH levels in RAS-mutant cancer cells. Inhibition of GPX4 results in an aggregation of iron-dependent lipid peroxide, which damages cell membranes and eventually leads to cell death without the usage of particular effector molecules as in other processes of programmed cell death [18]. Research has shown that iron chelation or lipophilic antioxidant could help avoid ferroptosis, but inhibitors of other forms of cell death did not work [28,29]. According to recent studies, ferroptosis is responsible for the pathological processes in a number of liver disorders, containing ischemia-reperfusion injury [30], NAFLD [31], and hepatocellular carcinoma [32]. In our study, typical characteristics of ferroptosis, including excessive iron levels and an increase in lipid peroxidation, were found in hepatocytes of iron-overloaded mice. Iron overload induced a robust increase in Ptgs2 and a significant reduction in GPX4, which are both putative molecular markers of ferroptosis. Thus, system Xc- is a cystine/glutamate antiporter in the cell membrane responsible for cellular cystine uptake to synthesize glutathione. Since cysteine is necessary for the production of the antioxidant GSH, iron overload led to a decrease in intracellular GSH and inactivation of GPX4, increased lipid ROS formation and peroxidation with increasing MDA, Ptgs2 and COX2, triggering ferroptosis.

In conclusion, this study demonstrated that iron overload inhibited lipogenesis and lipidoses and prevented body fat accumulation of mice even upon HFD. In addition, though insulin sensitivity was impaired, iron overload protected the liver from obesity-associated pathological conditions like hepatic steatosis. Moreover, RNA-seq analysis revealed ferroptosis induced by iron overload and exhibited a possible role in glucolipid metabolism in the liver. Our findings shed fresh light on the role of ferroptosis in lipid accumulation regulation. Iron overload leads to disorders of the body’s glucolipid metabolism and causes toxicity in organs such as the liver and heart. Therefore, we must strictly regulate the iron balance in the body. Due to the high iron content in red meat, it is recommended to reduce the intake of red meat.

## Figures and Tables

**Table 1:** Primer sequences applied in real-time quantitative PCR.

Gene	Forward Primer (5′-3′)	Reverse Primer (5′-3′)
Hamp	TTGCGATACCAATGCAGAAG	TGCAACAGATACCACACTGG
FtL	CACCTACCTCTCTCTGGGCT	CGCGATCGTTCTGAAACTCG
SREBF1	ACTTTTCCTTAACGTGGGCCT	CATCTCGGCCAGTGTCTGTT
ACACA	CTGTATGAGAAAGGCTATGTG	AACCTGTCTGAAGAGGTTAG
GLUT4	TGAAGATGAGTGTCCTGTGCTG	GAAGTGCAAAGGGTGAGTGAG
FAS	TCCAAGACTGACTCGGCTACTGAC	GCAGCCAGGTTCGGAATGCTATC
SCD1	GTGGGGTAATTATTTGTGACC	TTTTTCCCAGACAGTACAAC
PGC1α	TCCTCTTCAAGATCCTGTTA	CACATACAAGGGAGAATTGC
Cpt1α	GGGAGGAATACATCTACCTG	GAAGACGAATAGGTTTGAG
PPARα	AGTTCGGGAACAAGACGTTG	CAGTGGGGAGAGAGGACAGA
PLIN2	GACACCACCTGCATGGCT	TGAAGCAGGGCCACTCTC
PPARγ	TCTTCCATCACGGAGAGGTC	GATGCACTGCCTATGAGCAC
PCK1	AAGCGGATATGGTGGGAACTC	CTCCAAATTTCCTCCCAGGGT
Fbp1	AAGTACTGATGAGCCTTCTG	GCTCACCATAATGAATTCTCC
Pfk1	AAGAGACTGATTTTGAGCAC	CTCAGAAACCCTTGTCTATG
Pklr	GTGAAGAAGTTTGATGAGATCC	CAAGAAAACCTTCTCTGCTG
Slc7a11	CTTTGTTGCCCTCTCCTGCTTC	CAGAGGAGTGTGCTTGTGGACA
Ptgs2	GGGTTGCTGGGGGAAGAAA	CTCTGCTCTGGTCAATGGAGG
GPX4	CCTCCCCAGTACTGCAACAG	GGCTGAGAATTCGTGCATGG
FtH	TCAGTCACTACTGGAACTGC	CGTGGTCACCCAGTTCTTTA

## Data Availability

Data are contained within the article.
